# Blood Component Utilization and Discard Patterns in Telangana, India: A Three-Year Retrospective Study

**DOI:** 10.7759/cureus.99846

**Published:** 2025-12-22

**Authors:** Jeeth Rai, Nirlipta Kumar Behera, Manoj Kumar Gupta, Prathyusha Vemuri, Balaji Dhanraj Kendre, Heena Dixit, Rahul VC Tiwari, Deepak Agrawal, Seema Gupta

**Affiliations:** 1 Department of Periodontology, Bharati Vidyapeeth (Deemed to be University) Dental College and Hospital, Sangli, IND; 2 Department of Anaesthesiology, Fakir Mohan Medical College and Hospital, Balasore, IND; 3 Department of Pathology and Blood Bank, Mahamaya Rajkiya Allopathic Medical College, Ambedkar Nagar, IND; 4 Department of Prosthodontics, Care Dental College, Guntur, IND; 5 Department of Orthodontics, Saraswati Dhanwantari Dental College and Hospital, Parbhani, IND; 6 Department of Medical Health Administration, Index Medical College, Hospital and Research Center, Malwanchal University, Indore, IND; 7 Department of Oral and Maxillofacial Surgery, Narsinhbhai Patel Dental College and Hospital, Visnagar, IND; 8 Department of Orthodontics, Kothiwal Dental College and Research Centre, Moradabad, IND

**Keywords:** blood bank, plasma, red blood cells, retrospective, transfusion

## Abstract

Background

Blood transfusion services play a central role in emergency and routine clinical care, making the optimal utilization of blood components essential for healthcare efficiency. The present study aimed to evaluate statewide patterns of requisition, utilization, and discard of blood components across licensed blood banks in Telangana over a three-year period, and to identify component-specific and system-level factors contributing to wastage.

Methodology

This retrospective, observational study analyzed records from 50 licensed blood banks across Telangana between January 2022 and December 2024. Data on requisition, cross-matching, issue, transfusion, return, and discarding of packed red cells (PRCs), fresh frozen plasma (FFP), and platelet concentrates (PCs) were collected using a standardized protocol. Key indicators such as crossmatch-to-transfusion ratio (C/T), transfusion probability (%T), transfusion index (TI), utilization ratio, and discard ratio were calculated. Descriptive analyses were performed.

Results

Across the three-year period, approximately 51,063 units were issued, with an overall utilization ratio of 87.7% and a discard ratio of 12.3%. The annual discard ratio increased from 11.7% (2022) to 13.3% (2024). Component-wise, PRCs had the highest utilization (92.1%) and lowest discard (7.9%), whereas FFP and PCs showed lower utilization (~83%) and higher discard ratios (16.9% and 17.0%, respectively). Expiry was the leading cause of waste, rising from 1,068 units (2022) to 1,373 units (2024). The mean C/T ratio was 1.5, %T was 66.1%, and TI was 0.82, although there was notable interfacility variability. Intensive care and surgical units demonstrated the highest utilization, while oncology and pediatrics showed suboptimal performance.

Conclusions

Although red cell utilization in Telangana blood banks is efficient, the persistently high wastage of platelets and plasma and the increasing expiry-related discard trend highlight the need for improved inventory management, redistribution systems, and statewide patient blood management strategies.

## Introduction

Blood transfusion services form a vital lifeline in modern healthcare, supporting the management of trauma, major surgeries, obstetric emergencies, malignancies, and chronic anemia. With rising demand and persistent shortages, the rational use of blood and its components has become a global priority [1]. In low- and middle-income countries, despite a well-structured national blood policy advocating voluntary donation and component separation, significant gaps persist in clinical practices, leading to precautionary overordering, underutilization of issued units, and high discard ratios [2,3]. These inefficiencies not only escalate costs but also undermine the efforts of voluntary donors and strain an already limited resource.

The shift from whole blood to component therapy has improved therapeutic precision; however, it has introduced new challenges related to shelf life and storage conditions. Packed red blood cells (PRCs) enjoy a longer shelf-life of 35-42 days, whereas platelets remain viable for only five days, and fresh frozen plasma (FFP), although stable for one year when frozen, often faces expiry after thawing if not utilized promptly [4]. Consequently, platelets and plasma consistently record higher waste worldwide, particularly in resource-constrained settings with unpredictable demand and suboptimal inventory management [5].

In India, a previous single-center study reported a discard ratio of 6.95%, largely attributable to expiry, transfusion-transmitted infection (TTI) seropositivity, and clerical errors [6]. However, most audits are institution-specific, of short duration, or limited to whole blood, offering little insight into state-wide or component-specific trends. Telangana, with its rapidly expanding network of over 120 licensed blood banks serving urban, semi-urban, and rural populations, lacks comprehensive data to date on requisition patterns, utilization efficiency, and discarding causes across diverse healthcare tiers.

Therefore, this retrospective study was undertaken to analyze blood component requisition, issue, transfusion, and discard practices across 50 licensed blood banks in Telangana using standardized quality indicators such as crossmatch-to-transfusion ratio (C/T), transfusion probability (%T), and transfusion index (TI), with the aim of generating evidence-based insights to strengthen rational transfusion practices and minimize preventable wastage in the region.

## Materials and methods

This retrospective observational study was conducted over a three-month period (May-July 2025). The analysis encompassed data spanning three calendar years (January 1, 2022, to December 31, 2024) collected from 50 licensed blood banks across Telangana, India. The participating blood banks were purposively selected from the state’s 153 licensed facilities (as per Drugs Control Administration, Telangana, January 2025) to ensure representation of all healthcare tiers and geographical regions: tertiary-care teaching and corporate hospitals in urban areas, district and area hospitals in semi-urban locations, and community health centers with standalone blood banks in rural districts. Only centers equipped with component separation facilities and maintaining complete records throughout the study period were included; those exclusively providing whole-blood transfusions or having more than 10% missing data in any year were excluded.

A convenience sampling approach was employed, wherein licensed blood banks that were willing to participate and had complete records for the study period were included. To enhance representativeness and reduce selection bias, purposive stratification was applied to ensure inclusion of blood banks from different healthcare tiers (tertiary-care teaching and corporate hospitals, district hospitals, and rural standalone blood banks) and from diverse geographic regions of Telangana, including urban, semi-urban, and rural areas.

The study protocol was approved by the Institutional Ethics Committee of Index Medical College, Malwanchal University, Indore, Madhya Pradesh, India (IEC Ref: MU/RO/Ph.D/GN/2025/121), which served as the coordinating institution. Additional administrative approval was obtained from the Telangana State Blood Transfusion Council and medical superintendents or directors of individual centers. As the study involved only aggregate, anonymized data without patient or donor identifiers, the requirement for individual consent was waived.

All documented requirements for blood components, such as PRCs, FFP, and platelet concentrate (PC), issued during the study period were included, and records of requisition, cross-matching, issue, transfusion, return, and discard were complete. Whole-blood units, emergency uncross-matched O-negative issues without subsequent transfusion confirmation, autologous or directed donations, and records with ambiguous reasons for discarding were excluded.

Data were extracted using standardized, pre-tested proforma from blood bank registers, issue and transfusion logs, discard registers, and requisition forms. The variables collected included total units requisitioned, cross-matched, issued, transfused, returned unused, and discarded; component type; requesting clinical department; reason for discarding (expiry, TTI seroreactivity, leakage/hemolysis, clotting, broken bag, clerical/labeling error, or others); donor category (voluntary/replacement); and relevant dates. Four trained postgraduate residents performed data collection over four months, with 10% of the records independently verified by the principal investigator. Double-data entry was performed, and discrepancies were resolved by re-examination of the original registers.

Transfused probability (%T) was calculated as follows [7]:

\[ \text{Percent unit transfused (%T)} = \frac{\text{Patients transfused}}{\text{Patients cross-matched}} \times 100 \% \]

Acceptable T≥30%.

The transfused index (TI) was calculated as follows [7]:

\[ \text{Transfusion index (T)} = \frac{\text{Patients transfused}}{\text{Patients cross-matched}} \]

Utilization ratio was calculated as follows [7]:

\[ \text{Utilization ratio} = \frac{\text{Units transfused}}{\text{Units issued}} \times 100\% \]

Discard ratio was calculated as follows [7]:

\[ \text{Discard ratio} = \frac{\text{Units discarded}}{\text{Units issued}} \times 100\% \]

The primary outcomes were overall and component-specific utilization and discard ratios, along with the reasons for discarding. Secondary outcomes included C/T ratio [7], %T, and TI across departments and facility types, as well as year-wise trends over the three-year study period. Data were analyzed using SPSS version 23.0 (IBM Corp., Armonk, NY, USA). Descriptive statistics are expressed as frequencies, percentages, and means ± standard deviations. Year-wise comparisons were descriptive; no formal statistical test for temporal trend was applied. Comparisons across blood components and clinical departments were descriptive in nature; no inferential statistical testing was performed for these comparisons.

## Results

Table 1 summarizes the yearly utilization and discarding ratio of blood components from 2022 to 2024. The number of requisitioned and issued components shows a steady increase over the years, with the highest requisition in 2023. Although the number of transfusions has also increased, 2024 recorded a slight decline in utilization efficiency, reflected by the lowest utilization ratio of 86.7%. The discard ratio increased to 13.3% in 2024, indicating higher wastage compared to previous years. Overall, across the three years, an average utilization ratio of 87.7% and a discard ratio of 12.3% highlight consistent service efficiency, with a need for improved inventory management to reduce discards.

**Table 1:** Annual trends in requisition, issue, transfusion, and discard of blood components over three consecutive years, along with calculated utilization and discard ratios.

Year	Components requisitioned	Components issued	Components transfused	Components discarded	Utilization ratio (%)	Discard ratio (%)
2022	18,209	16,571	14,638	1,933	88.3%	11.7%
2023	19,142	17,316	15,267	2,049	88.2%	11.8%
2024	19,131	17,176	14,884	2,292	86.7%	13.3%
Total	56,482	51,063	44,789	6,274	87.7%	12.3%

Table 2 presents the utilization and discard ratio of different blood components. PRC showed the highest efficiency, with a utilization ratio of 92.1% and the lowest discard ratio of 7.9%. In contrast, FFP and PC demonstrated lower utilization ratios and higher discard ratios. These findings indicate that PRCs are used more effectively, whereas FFP and PCs experience greater wastage, likely due to their shorter shelf life and specific storage requirements.

**Table 2:** Component-wise efficiency metrics across the study period.

Component type	Requisitioned	Issued	Transfused	Discarded	Utilization ratio (%)	Discard ratio (%)
Packed red blood cells	28,127	26,048	24,002	2,046	92.1%	7.9%
Fresh frozen plasma	15,764	13,721	11,409	2,312	83.1%	16.9%
Platelet concentrates	12,591	11,294	9,378	1,916	83.0%	17.0%
Total	56,482	51,063	44,789	6,274	87.7%	12.3%

Table 3 shows the department-wise utilization of the blood components. The intensive care unit (ICU) demonstrated the highest utilization ratio (94.5%), followed closely by the surgery department (92.3%), indicating the efficient use of blood units. Obstetrics and gynecology (89.6%) and medicine (88.2%) also showed good utilization. Oncology recorded a moderate rate of 78.8%, while the “Others” category, including pediatrics, had the lowest utilization at 75.6%. Overall, across all departments, the utilization ratio was 87.7%, reflecting the generally effective use of blood components.

**Table 3:** Departmental comparison showing efficiency of blood component use. “Others” includes Pediatrics, Neonatology, Emergency Medicine, and other low-volume departments.

Department	Components issued	Components transfused	Utilization ratio (%)
Surgery	14,441	13,329	92.3
Obstetrics & Gynecology	9,292	8,326	89.6
Medicine	10,192	8,989	88.2
Oncology	7,261	5,722	78.8
Intensive Care Unit	5,104	4,823	94.5
Others (Pediatrics, etc.)	4,772	3,608	75.6
Total	51,063	44,797	87.7

Table 4 summarizes the annual reasons for discarding blood components from 2022 to 2024. Expiry remained the most common cause, steadily increasing from 1,068 units in 2022 to 1,373 units in 2024 and contributing to more than half of all discards. TTI seropositivity was the second major reason, followed by leakage or breakage, both of which showed a gradual increase over the years. Clerical errors and clot formation accounted for smaller but consistent proportions of discarded units. Overall, 6,274 components were discarded across three years, indicating the need for improved inventory management, enhanced donor screening, and stricter handling protocols to reduce preventable wastage.

**Table 4:** Reasons for discard of blood components by year, n (%). n = number of discarded items. TTI: transfusion-transmitted infection

Discard reason	2022 (n = 1,933)	2023 (n = 2,049)	2024 (n = 2,292)	Total (n = 6,274)
Expiry	1,068 (55.3)	1,194 (58.3)	1,373 (59.9)	3,635 (58.0)
TTI seropositivity	445 (23.0)	470 (22.9)	492 (21.5)	1,407 (22.4)
Leakage/Breakage	193 (10.0)	203 (9.9)	263 (11.5)	659 (10.5)
Clerical errors	145 (7.5)	155 (7.6)	165 (7.2)	465 (7.4)
Clot formation	82 (4.2)	85 (4.1)	90 (3.9)	257 (4.1)
Total	1,933 (100)	2,049 (100)	2,292 (100)	6,274 (100)

Table 5 presents the operational efficiency indicators for various blood banks. BB-01 showed the highest efficiency, with the lowest C/T ratio (1.21), highest transfusion probability (82.4%), and a strong transfusion index (0.82). Blood banks BB-07, BB-14, and BB-29 demonstrated lower efficiency, as reflected by higher C/T ratios and reduced transfusion probability. BB-21, BB-37, and BB-45 showed moderate to good performance, with balanced utilization indicators. The mean C/T ratio of 1.5 and transfusion probability of 66.1% suggested overall acceptable efficiency across centers, although variability indicated by the standard deviations highlights the need for performance standardization and improved crossmatch-to-transfusion practices. Table 5 presents selected examples to demonstrate variability in performance indicators across blood banks; summary statistics derived from this subset are not intended to represent statewide estimates.

**Table 5:** Performance metrics of participating blood banks (BBs) using standard transfusion indices.

Blood bank code	Units crossmatched (C)	Units transfused (T)	C/T ratio	Transfusion probability (%T)	Transfusion index (TI)
BB-01	2,410	1,986	1.21	82.4	0.82
BB-07	1,893	1,102	1.72	58.2	0.58
BB-14	1,504	802	1.87	53.3	0.53
BB-21	2,208	1,576	1.40	71.4	0.71
BB-29	1,601	904	1.77	56.5	0.56
BB-37	1,946	1,365	1.42	70.1	0.70
BB-45	1,701	1,209	1.41	71.1	0.71
Mean	1,894.7	1,277.7	1.50	66.1	0.70
SD	327.21	408.21	0.24	10.4	0.10

## Discussion

This three-year retrospective study involving 50 licensed blood banks in Telangana provides the most comprehensive statewide insight into blood component utilization and wastage patterns in a rapidly developing Indian state. During the study period (2022-2024), approximately 51,063 blood component units were issued, with 87.7% utilization ratio and 12.3% discard ratio. Kulkarni et al. [8] found that a total of 3,280, 1,868, and 486 units of whole blood were collected in 2018, 2019, and 2020, respectively. The observed whole blood discard ratios were 9.48%, 17.23%, and 43% in 2018, 2019, and 2020, respectively, in a tertiary care hospital blood bank in North Karnataka. A progressive increase in the discard ratio from 10.8% in 2022 to 13.3% in 2024 was found in our study despite increasing requirements, signals worsening inventory-pressure mismatch, and calls for urgent system-level corrective measures.

Component-specific analysis reinforces global trends: PRC exhibited the highest utilization (92.1%) and lowest wastage (7.9%), attributable to its longer shelf-life (35-42 days) and more predictable demand. Far et al. [9] determined that a significant proportion (77.9%) of PRCs were rendered unusable owing to expiration, with a reported variability ranging from 1.93% to 30.7%. The rates of wastage for PRC, FFP, and PC were quantified as 5.7 ± 0.7, 1.4 ± 0.4, and 3.2 ± 0.5, respectively, as documented by Amini Kafi-Abad et al. [10] in their study conducted in Iran between the years 2005 and 2015. The principal factors contributing to the wastage of blood components were identified as expiration and protocols surrounding the reservation and return of these components from the operating room.

In contrast, platelets (random donor PC or pooled PC) and FFP recorded utilization ratios of only ~83% and discard ratios exceeding 16%, findings almost identical to large audits from India (platelets 51.8%) [11] and New Hampshire (plasma 43% to 66%) [12]. The short shelf life of platelets (five days) and narrow post-thaw window for FFP (24 hours at 4°C) make them highly vulnerable to outdating in settings with fluctuating emergency demand and limited real-time inter-facility transfer mechanisms, a challenge repeatedly documented in low- and middle-income countries [1].

The predominant reason for discarding across all years was expiry, followed by TTI seroreactivity. The rising absolute number of expiry-related discards despite increasing collections reflects overstockpiling driven by fear of shortages, a behavioral pattern well-described in transfusion medicine literature as “just-in-case” ordering [13]. Suresh et al. [14] reported that a 49% discard ratio was due to TTI, suggesting scope for further improvement in donor questionnaire sensitivity, pre-donation counseling, and adoption of pathogen reduction technologies where feasible.

Department-wise utilization data offers actionable insights. ICUs and surgical departments achieved near-optimal efficiency, likely reflecting protocol-driven transfusion triggers and bedside decision making by senior clinicians. Conversely, oncology and miscellaneous (including pediatric) departments exhibited significantly lower utilization, consistent with reports of prophylactic platelet transfusions at higher thresholds and overuse of FFP in hypoalbuminemia or liver disease without coagulopathy. Similar findings have been reported in previous studies [15,16]. These practices contravene international patient blood management (PBM) guidelines and contribute disproportionately to platelet and plasma wastage [17].

The mean C/T ratio of 1.5, %T of 66.1%, and TI of 0.82 are considerably better than earlier Indian studies reporting C/T ratios of 1-2.3 [18,19] and approach the benchmarks recommended by the British Committee for Standards in Haematology (C/T ≤2.0, %T ≥50%) [20]. However, the wide inter-facility variation (C/T ranging from 1.21 to >3.0) highlights the unequal adoption of maximum surgical blood ordering schedules, type-and-screen policies, and electronic crossmatch systems. Centers achieving C/T ratios close to 1.0, such as tertiary teaching or corporate hospitals with 24×7 specialist cover and robust PBM programs.

Clinical and policy implications

The 12-13% discard ratio corresponds to approximately 6,274 wasted components over three years, underscoring the substantial operational and potential economic impact of blood component wastage at the system level. More importantly, this undermines public trust in voluntary donations. Implementing statewide real-time inventory dashboards, first-in-first-out (FIFO) strict enforcement, daily platelet/FFP pooling and redistribution networks, and mandatory prospective audit of all FFP and prophylactic platelet requests could reduce wastage. Adoption of single-unit PRC policies in stable anemia, restrictive thresholds (hemoglobin <7-8 g/dL), and viscoelastic testing in place of conventional coagulation screens would further rationalize their use without compromising outcomes [21].

Limitations

The study relied on retrospective register-based data; underreporting of returned or discarded units cannot be ruled out. Emergency O-negative uncross-matched issues and whole-blood transfusions were excluded, potentially underestimating the total wastage in rural facilities. Detailed clinical indications and patient outcome data were not captured, limiting the correlation with inappropriate use. Finally, although centers were purposively selected for representation, participation was restricted to those with component separation facilities and complete records, introducing a degree of selection bias toward better-resourced blood banks.

## Conclusions

This multicenter study revealed that Telangana’s blood transfusion services achieved high red cell utilization and an acceptable mean C/T ratio; however, the overall component wastage reached 12.3%, driven predominantly by expiry of platelets and FFP. The rising discard trend over three years, despite increasing collections, persistent over-ordering, and inadequate redistribution mechanisms. Targeted implementation of statewide real-time inventory sharing, strict FIFO protocols, restrictive transfusion thresholds, and robust PBM programs can substantially reduce preventable waste, enhance resource efficiency, and strengthen public trust in voluntary blood donation across the region.
